# Iatrogenic atrial septal defect following transcatheter edge-to-edge mitral valve repair: A systematic review and meta-analysis

**DOI:** 10.1097/MD.0000000000046486

**Published:** 2025-12-26

**Authors:** Deng Lei, Chunlan Zhou, Junqing Zhou, Chen Luo, Baoshi Zheng

**Affiliations:** a Department of Cardiac Surgery, Shaoxing People’s Hospital, Shaoxing, China; b Department of Radiology, Shaoxing People’s Hospital, Shaoxing, China; c Department of Cardiac Surgery, the First Affiliated Hospital of Guangxi Medical University, Nanning, China.

**Keywords:** iatrogenic atrial septal defect, meta-analysis, mitral regurgitation, systematic review, transcatheter edge-to-edge mitral valve repair

## Abstract

**Background:**

Transcatheter edge-to-edge mitral valve repair (TEER) requires transseptal access of the left atrium, which usually causes an iatrogenic atrial septal defect (iASD). It is controversial that whether these patients with iASD persistence will progress to a bad prognostic state. So it’s worthwhile to evaluate whether the procedure of TEER induced iASD leads to development of a relevant interatrial shunt. This systematic review and meta-analysis aimed to identify predictors of iASD persistence in patients with mitral regurgitation (MR) undergoing TEER and assess its effect on in-hospital outcomes and mortality.

**Methods:**

In this systematic review and meta-analysis, we conducted a literature search in various databases. Our investigation summarized data from contemporary studies on the incidence of iASD following TEER for MR and its association with clinical outcomes.

**Results:**

10 studies were analyzed, possible predictors of risk factors for the persistence of iASD after TEER seemed to be atrial fibrillation before surgery (OR 1.40; *P* = .04), residual MR > 2^+^(OR 2.36; *P* = .001), residual tricuspid regurgitation > mild(OR 1.52; *P* = .01), and prolonged fluoroscopic time(MD [in minutes] 5.36; *P* = .007). Patients with iASD persistence after TEER seemed to have a greater risk to suffer from right heart overload reflecting from the enlarged right ventricle end-diastolic dimension (RVEDD) (MD 3.27; *P* < .00001) and the enlarged diameter of the right atrium (MD 4.41; *P* < .00001). Patients with iASD persistence after TEER had a greater risk of heart failure rehospitalization (OR 2.71; *P* = .003).

**Conclusion:**

This systematic review and meta-analysis identified several related predictors of iASD persistence, possibly leading to right heart volume overload and a higher risk of heart failure rehospitalization. In order to generalize our findings, larger clinical studies in independent patient cohorts are necessary. What’s more, there is a need to perform a careful decision for interventional closure of an iASD after TEER.

## 1. Introduction

Mitral regurgitation (MR) is the most prevalent valvular heart disease among the elderly, with an increasing incidence as age increases. The incidence of severe MR among people over 65 years old is as high as 19.1%, and it is estimated that 7.5 million MR patients in China will need interventional treatment by 2025.^[1]^ Surgery is the main way to treat severe MR at present, but more than 2/3 of patients cannot undergo surgery due to old age, complications, and other risk factors, the 5-year mortality rate is as high as 50%.^[2]^ Transcatheter mitral edge-to-edge repair (TEER) alleviates MR symptoms in patients with high surgical risk by clamping the anterior and posterior valves of the regurgitant mitral valve, it provides a new treatment option for severe MR and episodes of acute MR.^[3]^ Reduction of MR has been shown to improve hemodynamics by reducing volume overload, atrial filling pressures, and pulmonary filling pressures, and further increasing forward stroke volume with succeeding improvement of functional status and clinical outcome.^[4]^ Previous studies have shown that TEER with Mitraclip is able to reduce MR effectively in patients with atrial functional MR and seems to confer more clinical and hemodynamic benefits, including the reduction in systolic pulmonary artery pressure and higher cardiac output than in ventricular functional MR.^[5]^ Since 2003, when the Mitraclip system was used for the first time in the world to complete TEER, more than 150,000 cases have been performed worldwide by 2021. With the rapid development of TEER, it has become a frontier domain in the interventional therapy of structural heart disease, and has been widely used and recommended by guidelines around the world.^[6]^ Atrial septal puncture is the most critical step at the beginning of TEER, which needs to be carried out under the guidance of intraoperative transesophageal echocardiography. Ideal puncture results can reduce the difficulty of operation, the operating time and the risk of complications.^[7]^ Despite increasing understanding of the specific procedural characteristics and technical aspects of TEER, the effect of iatrogenic atrial septal defect (iASD) on clinical outcomes in patients after TEER is not fully understood. because persistent iASD is associated with worse hemodynamic and clinical progression after TEER, and more importantly-with increased overall mortality. It’s necessary to investigate further studies if persistent iASD is the mediator of advanced diseases or complications in these patients after TEER. In this background, we sought to systematically review the literature and perform a comprehensive meta-analysis in order to provide a quantitative assessment of evidence about the impact of iASD on mortality and in-hospital outcomes as well as identify predictors for iASD in patients after TEER.

## 2. Methods

This systematic review was conducted in accordance with the Preferred Reporting Items for Systematic Reviews and Meta-Analyses (PRISMA) guidelines and in line with the protocol agreed by all authors. A systematic search of the literature was performed in PubMed, MEDLINE, EMBASE, Cochrane Library, Web of Science, and the Cochrane Central Register of Controlled Trials from database inception up to the final search date of June 11, 2024. The keywords were listed such as (atrial septal defect OR ASD) AND (mitral valve repair OR edge-to-edge OR MVR OR MitraClip). In addition, the reference lists of relevant papers were screened (backward snowballing). Institutional review board approval and patient consent were not required because of the nature of this systematic review and meta-analysis. Eligible studies met the following PICOS criteria: Population: Adult human patients with MR who underwent TEER; Intervention: Patients who developed iASD following TEER; Comparative intervention: Patients who did not develop iASD following TEER; Outcome: Any outcome of the present meta-analysis (reported below); and Study design: Prospective and retrospective studies as well as randomized control trials. Two investigators (C.L.Z. and J.Q.Z.) independently executed the search and evaluated the study eligibility and study quality, discrepancies were resolved by consensus with the addition of a third reviewer (C.L.). Data on study characteristics, patient and procedural characteristics, and outcomes were extracted using data collection sheets. The prespecified primary endpoints were the composite of overall mortality or HF rehospitalization. Secondary endpoints were the New York Heart Association (NYHA) functional class III or IV at follow-up and stroke/transient ischemic attack (TIA). Moreover, predictors of iASD persistence following TEER were also identified and meta-analyzed. The Newcastle Ottawa Scale was also used to assess the quality of evidence of the included studies. Pooled odds ratios (ORs) for dichotomous data and mean difference (MD) for continuous data with corresponding 95% confidence interval (CI) were calculated. Whenever applicable, mean ± SD was calculated by median and interquartile range.^[8]^ Statistical heterogeneity was assessed through the Cochran Q statistic and *I*^2^ values. *I*^2^ values of <25%, 25% to 50%, or >50% were indicative of low, moderate, or high heterogeneity, respectively.^[9]^ Publication bias and small study effect were assessed by visual inspection of funnel plots and using Egger’s test. A leave-one-out sensitivity analysis was conducted to show how each study might affect the overall estimate. Statistical significance was set at *P* < .05 (2-sided). Statistical analysis was performed with RevMan version 5.3.

## 3. Results

**Figure 1.**
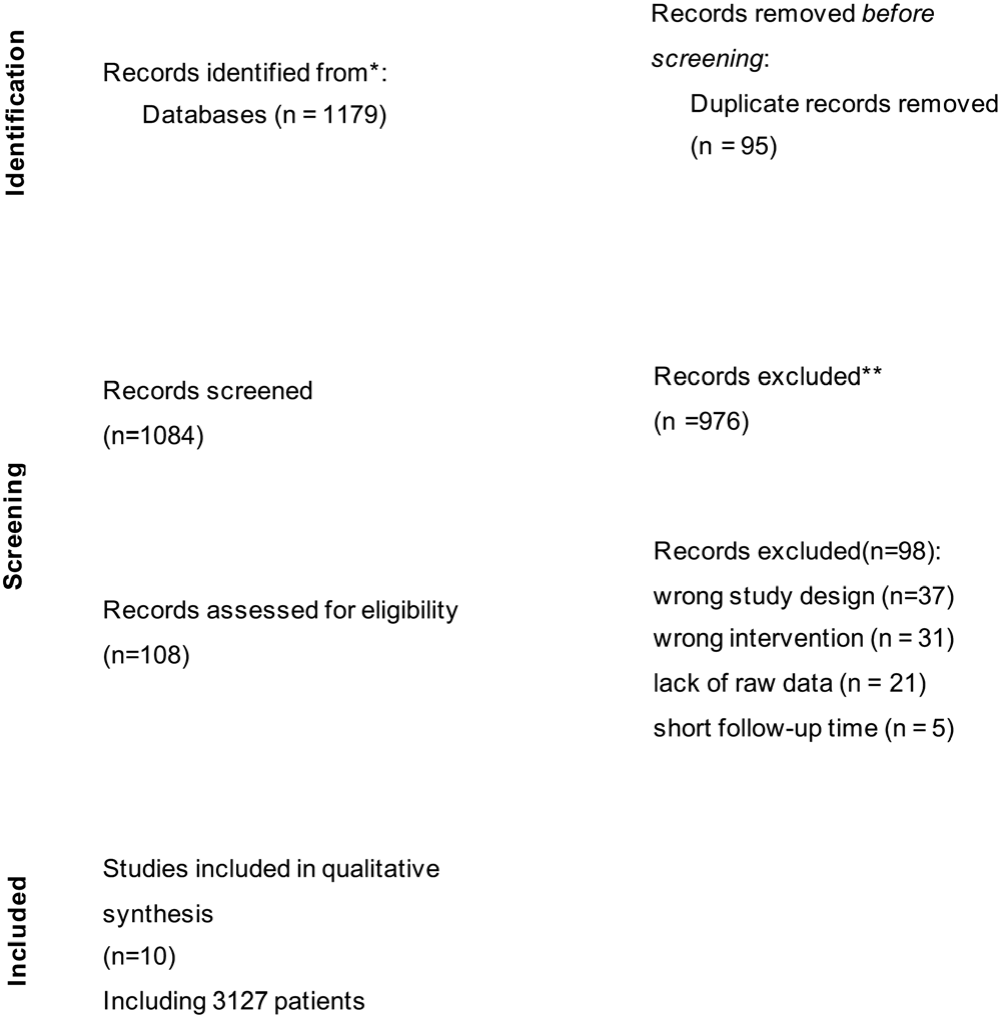
Flow diagram for study selection. Flow chart summarizing number of studies screened, assessed for eligibility, and included in the review, with reasons for exclusions at each stage.

The study flowchart is illustrated in Figure 1. A total of 1 randomized controlled trial and 9 observational studies were found to be eligible for inclusion in our meta-analysis. A total of 1179 studies were identified; after removing 95 duplicates, there were 1084 studies were screened by reading through the title and abstract sections, of which 108 were screened by reading through full text. Finally, 10 studies were identified and in accord with the inclusion criteria for meta-analysis. Among them, it was divided into 2 subgroups in study of Ikenaga et al,^[10]^ because 5 patients only completed the 1-month follow-up. For a summary of iASD prevalence at different follow-up times in Table 1, in which the essential features of included studies are listed. All studies were performed in Europe and USA and the follow-up duration ranged from 1 to 12 months. The baseline characteristics of the study populations are listed in Table 2. The proportion of female patients accounts for 50.1%. The prevalence of arterial hypertension (76.0%), diabetes mellitus (25.9%), chronic peripheral artery disease (25.9%), Stroke (8.73%), atrial fibrillation (AF, 59.3%), coronary artery disease (56.2%), and NYHA (III or IV) (78.1%) was high assuming patients with multimorbidity. We divided patients into iASD group and non-iASD group.

**Table 1:** Study characteristics.

Study	Region	Years of enrollment	Participants	iASD prevalence n (%)			
				1	6	9	12 (mo)
Smith 2012	USA	/	30	13 (43.3)	8 (26.7)	n/a	8 (26.7)
Paukovitsch 2021	Italy	2017–2018	48	n/a	n/a	n/a	20 (41.7)
Chao 2023	USA	2014–2020	316	n/a	n/a	108 (34.2)	n/a
Alachkar 2021	Germany	2014–2015	53	n/a	33 (62.3)	n/a	n/a
Ussia 2014	Italy	2012–2013	28	22 (81.0)	n/a	n/a	n/a
Schueler2015	Germany	/	66	n/a	33 (50.0)	n/a	n/a
Toyama 2017	USA	2007–2013	96	n/a	n/a	n/a	23 (24.0)
Ikenaga 2018	USA	2010–2014	131	74 (56.5)	n/a	n/a	24 (34.7)
Almalla 2017	Germany	2017	39	n/a	22 (56.4)	n/a	n/a
Blazek 2023	Germany	2016–2019	36	n/a	30 (83.3)	n/a	n/a
	Germany	2016–2020					

iASD = iatrogenic atrial septal defect.

**Table 2:** Baseline characteristics of included patients.

Study	Study type	Imaging modality	Age (yr)	Man (%)	BMI (kg/m^2^)	COPD (%)	Hypertension (%)	Pulmonary hypertension (%)	Dyslipidemia (%)	Diabetes mellitus (%)	Chronic kidney diseases (%)	Smoking (%)	Functional MR (%)	Degenerative MR (%)	NYHA (III or IV) (%)	CABG (%)	CAD (%)	Atrial fibrillation (%)	Stroke (%)	MI (%)	Peripheral artery disease (%)	STS score	EuroSCORE II
Schueler 2015	Prospective single-center study	TEE	77.1 ± 7.9	51 (76.7)	26.2 ± 4.2	/	47 (72)	/	28 (42)	18 (27)	/	24 (37)	48 (73)	/	/	/	40 (60)	46 (70)	3 (5)	/	/	/	10.1 ± 6.1
Paukovitsch 2021	Prospective single-center study	TEE	75.3 ± 8.5	23 (47.9)	27.3 ± 4.1	5 (10.4)	40 (83.3)	12 (25.0)	34 (70.8)	15 (31.3)	/	/	32 (66.7)	/	/	/	34 (70.8)	31 (64.6)	3 (6.2)	/.	/	4.2 ± 4.0	5.9 ± 5.6
Chao 2023	Prospective multi-center study	TTE	79.1 ± 9.1	102 (32.3)	27.2 ± 5.3	/	276 (87.3)	/	/	77 (24.4)	10 (3.2)	8 (2.5)	/	269 (85.1)	/	97 (30.7)	/	211 (66.8)	35 (11.1)	/	85 (26.9)	7.5 ± 5.5	/
Alachkar 2021	Prospective single-center study	TEE	76 ± 9	30 (57.0)	26 ± 6	19 (35)	40 (75)	/	/	21 (39)	33 (62)	/	21 (75)	10 (19)	48 (91)	/	38 (71)	42 (79)	/	/.	10 (18).	/	/
Ussia 2014	prospective observational study	/	74 ± 8	19 (68.0)	/	15 (53)	/	/	/	/	10 (36)	/	/	7 (25)	26 (92)	11 (39)	/	12 (43)	0	0	15 (53)	/	11.6 ± 7.3
Smith 2012.	Prospective single-center study	/	/	/	/	/	/	/	/	/	/	/	10 (33.3)	/	/	/	/	/	/	/.	/	/	/
Toyama 2017	A single center retrospective study	TTE	75.7 ± 12.2	58 (60)	/	/	73 (76)	/	/	28 (29)	/	/	59 (61)	/	/	36 (38)	58 (60)	55 (57)	4 (4)	24 (25)	/	/	/
Ikenaga 2018	A single center retrospective study	TTE	76 ± 16	80 (61.1)	24 ± 4.	22 (16.8)	90 (68.7)	/	76 (58)	30 (22.9)	21 (16)	/	71 (54.2)	/	77 (80)	/	71 (54.2)	58 (44.3)	18 (13.7)	/	/	10.3 ± 8.1	/
Almalla 2017	Prospective single-center study	TEE	75 ± 9	20 (51.2)	/	/	/	/	/	/	/	/	/	/	117 (89.3)	/	/	/	/	8 (22.2)	/	10.5 ± 4.5	24 ± 12
Blazek 2023	RCT (NCT03024268)	3D TEE.	73 ± 8	23 (63.9)	26.5 ± 4.5	6 (16.7)	32 (88.9)		28 (77.8)	13 (36.1)	/	/	23 (64)	13 (36)	/	5 (13.9)	/	25 (69.4)	3 (8.3)	/	2 (5.6)	/	6.5 ± 4.3

BMI = body mass index, CABG = coronary artery bypass grafting, CAD = cerebral arterial diseases, COPD = chronic obstructive pulmonary disease, RCT = randomized controlled trial, TEE = transesophageal echocardiography, TTE = transthoracic echocardiography.

**Figure 2.**
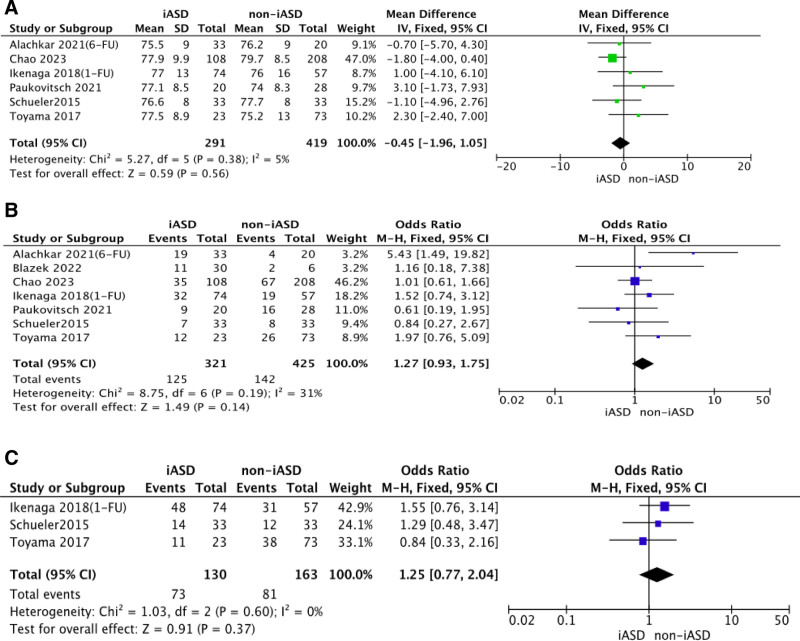
Forest plots for patient’s age, gender and number of clips implanted. Pooled mean difference for patient’s age (A), Pooled odds ratio for patient’s gender (B) and number of clips implanted (C), respectively, SD = standard deviation.

**Figure 3.**
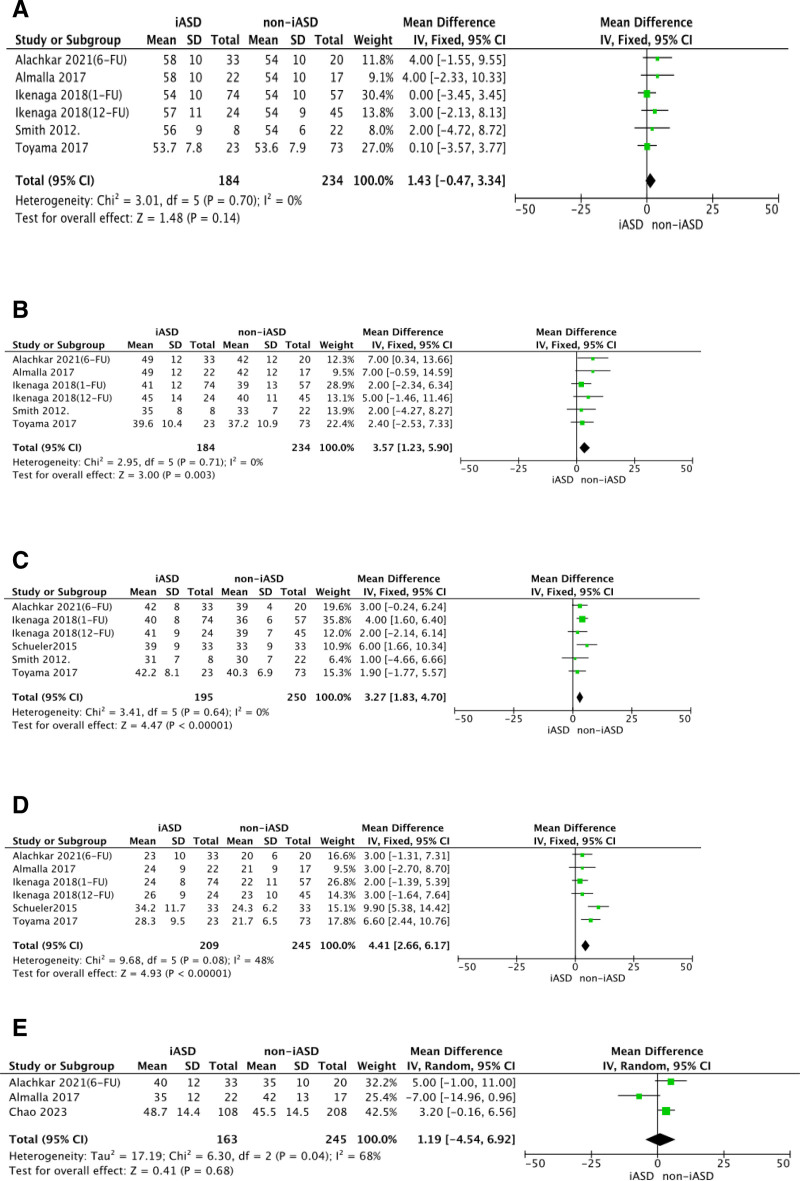
Forest plots for RVEDD, LVESD, RA area, RVSP, and LVEDD. Pooled mean difference for LVEDD (A), LVESD (B), RVEDD (C), RA area (D), and RVSP (E), respectively. LVEDD = left ventricular end-diastolic diameter, LVESD = left ventricular end-systolic diameter, RA area = right atrium area, RVEDD = right ventricle end-diastolic dimension, RVSP = right ventricular systolic pressure, SD = standard deviation.

By analyzing the preoperative data of the two groups, we could learn that there was no significant difference in patient’s age, gender, number of clips implanted between the iASD group and the non-iASD group. The results of the studies are listed in Figure 2. In addition, patients in the iASD group had bigger right ventricle end-diastolic dimension (RVEDD), left ventricular end-systolic diameter, and right atrium (RA) area, but lower left ventricular ejection fraction (LVEF). Patients with iASD persistence after TEER seemed to have a greater risk to suffer from right heart overload reflecting from the enlarged RVEDD (MD 3.27; *P* < .00001) and the increased RA area (MD 4.41; *P* < .00001). The results of the studies are listed in Figure 3.

**Figure 4.**
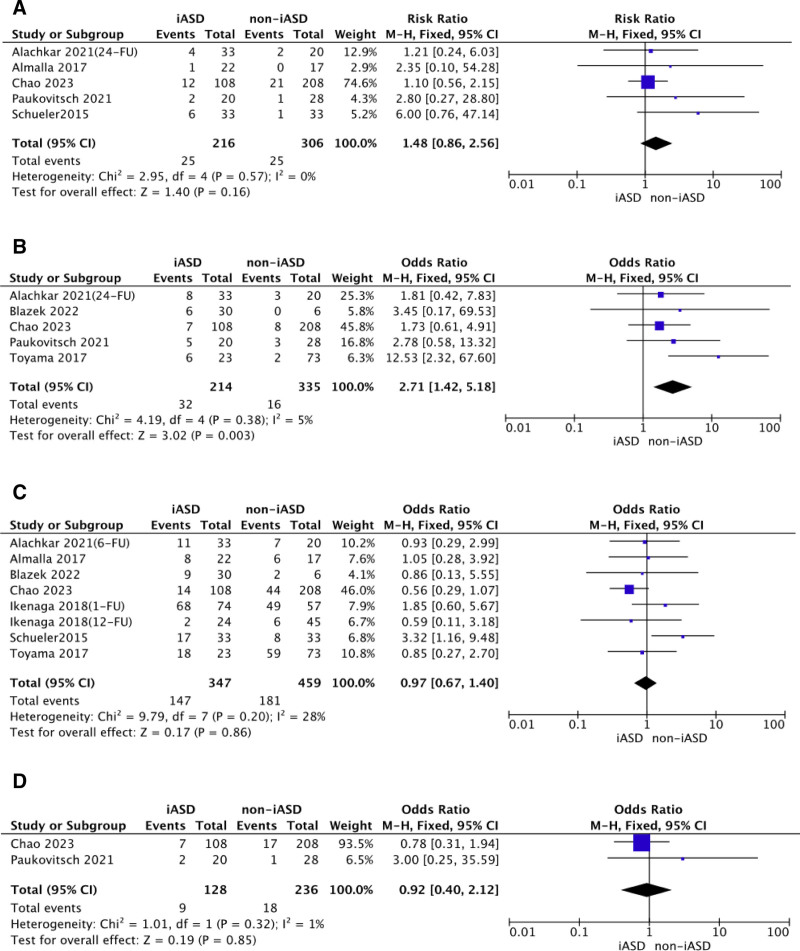
Patients’ clinical outcome. The evaluation of pooled OR for iASD persistence following TEER regarding patients’ clinical outcomes in Forest plot. iASD = iatrogenic atrial septal defect, NYHA = New York Heart Association, OR = odds ratios, TEER = transcatheter mitral edge-to-edge repair, TIA = transient ischemic attack.

**Figure 5.**
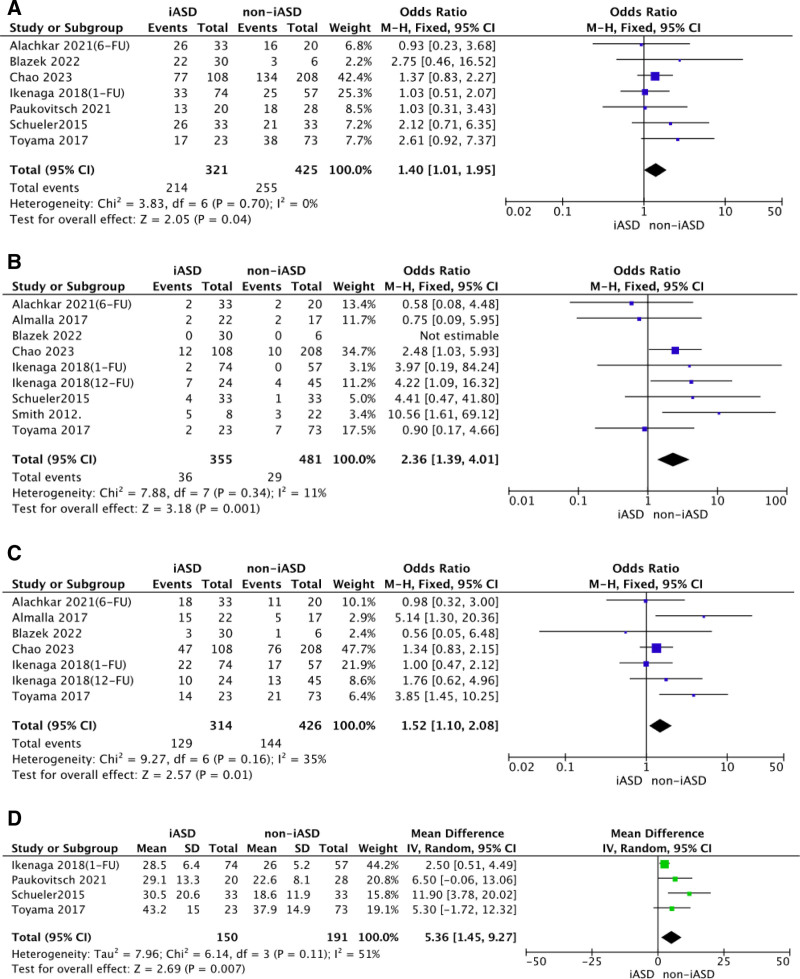
The predictors of persistent iASD in patients. The summary of pooled OR and MD for the predictor of persistent iASD, respectively, in Forest plot. AF = atrial fibrillation, iASD = iatrogenic atrial septal defect, MD = mean difference, MR = mitral regurgitation, OR = odds ratios, SD = standard deviation.

Primary endpoints were the composite of overall mortality or HF rehospitalization. Secondary endpoints were the NYHA functional class III or IV at follow-up and stroke/TIA. A case of rehospitalization was more possible to happen in patients with iASD persistence; the presence of iASD after TEER was associated with an increased risk of HF rehospitalization (OR 2.71, 95% CI: 1.42, 5.18, *P* = .003), with a low degree of heterogeneity (*I*^2^ 5%). There were no differences in overall mortality (OR 1.48, 95% CI: 0.86, 2.56, *P* = .16), NYHA functional class III or IV at follow-up (OR 0.97, 95% CI: 0.67, 1.40, *P* = .86) and stroke/TIA (OR 0.92, 95% CI: 0.40, 2.12, *P* = .85) between iASD patients and non-iASD patients after TEER (Fig. 4). What was noteworthy was that patients with iASD persistence had a high risk for MR grade >2^+^ at follow-up (OR: 2.36; 95% CI: 1.39, 4.01, *P* = .001), with a low degree of heterogeneity (*I*^2^ 23.7%). Patients with iASD persistence had a high risk for residual tricuspid regurgitation > mild at follow-up (OR: 1.52; 95% CI: 1.10, 2.08, *P* = .01), with a low degree of heterogeneity (*I*^2^ 35%). Likewise, significantly difference was noted in fluoroscopic time. Patients with iASD after TEER was associated with prolonged fluoroscopic time (mean difference [in minutes]: 5.36; 95% CI: 1.45, 9.27, *P* = .007), with high risk for heterogeneity (*I*^2^ 51%). It showed that possible predictors of risk factors for patients with iASD persistence after TEER seemed to be AF before surgery, residual MR > 2^+^, residual tricuspid regurgitation > mild, and prolonged fluoroscopic time, as shown in Figure 5.

**Figure 6.**
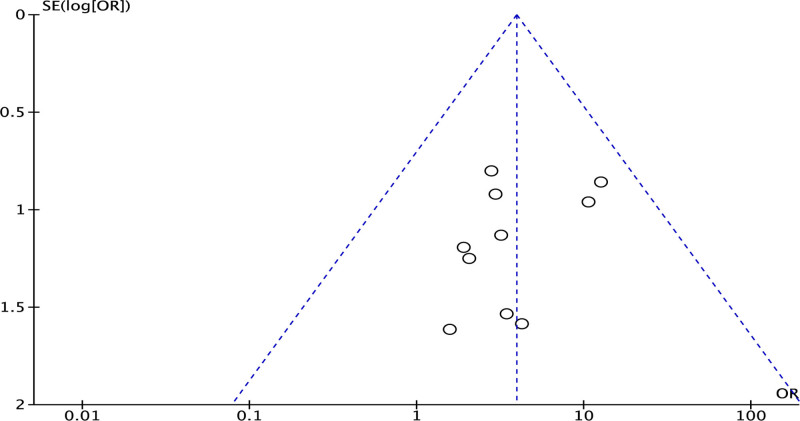
The Funnel plot of publication bias. OR = odds ratios.

We found that there was significant heterogeneity among the fluoroscopic time (*I*^2^ > 50%, *P* < .05). A sensitivity analysis of fluoroscopic time was performed by using the leave-one-out approach. When the reference was excluded, the heterogeneity decreased from 51% to 0%, indicating that the heterogeneity derived from this study. After the exclusion, the fixed-effect model was selected for further analysis, and the results showed that the difference was statistically significant (mean difference [in minutes]: 7.48; 95% CI: 3.35, 11.61; *P* = .0004). Similarly, there was significant heterogeneity among the right ventricular systolic pressure (*I*^2^ = 68%, *P* = .68), we used the leave-one-out approach to perform a sensitivity analysis. When the Almalla’s study was excluded, the heterogeneity decreased from 68% to 0%, indicating that the heterogeneity derived from this study, then the fixed-effect model was used for further analysis, and the results showed that the difference was statistically significant (MD: 3.63; 95% CI: 0.70, 6.56, *P* = .02). Funnel plot was used to evaluate the publication bias of the included literature, and no significant asymmetry was found, indicating no significant publication bias, as shown in Figure 6.

## 4. Discussion

TEER has emerged as a therapeutic option for high-risk patients with severe MR who are not suitable for surgical mitral valve repair or replacement,^[11,12]^ Incidence rate of iASD-persistence after TEER is relatively high in this patient population. However, during the era of TEER treatment with trans-septal approaches, the influencing risk factors for iASD-persistence, the functional consequences, and the effects on outcomes are not entirely clear.

The present systematic review and meta-analysis summarized data from contemporary studies on the incidence of iASD following TEER for MR and its association with clinical outcomes. The main findings of this study can be summarized as follows:

1. iASD persistence following TEER occurs in more than 20% of cases during 12 months follow-up, and iASD persistence seems to decrease over time.
2. Post-TEER iASD was associated with increased risk of HF rehospitalization and MR grade > 2^+^ at follow-up, as well as prolonged fluoroscopic time.
3. No differences were detected in terms of functional status (NYHA class III/IV) at follow-up, overall mortality, and stroke/TIA in patients with iASD persistence after TEER.
4. The echocardiographic parameters included for LVEF, left ventricular end-systolic diameter, RA area and RVEDD were significant different in patients between iASD and non-iASD persistence after TEER and several echocardiographic findings including RA area and RVEDD predict a greater risk to suffer from right heart overload in patients with iASD persistence after TEER.
5. Preexisting AF, prolonged fluoroscopic time, and residual mild tricuspid regurgitation appeared to predict a high risk to suffer from iASD persistence after TEER.

### 4.1. Prevalence and predictors of iASD after TEER

In previous studies, the high risk factors for the prevalence of iASDs after TEER procedure include catheter size, iASD-diameter, mitral valve calcification, higher degree residual MR, residual MR, longer time of the procedure, implanting multiple clips, and improper manipulation of the interatrial septum.^[13–16]^ Moreover, the high risk factors for the iASD-prevalence of other trans-septal procedures should be taken into account.

So far, it’s not yet completely clear about the predictors of iASD-persistence. We can draw some insights from certain echocardiographic parameters which are significantly different between + iASD patients and no-iASD patients, our studies suggest that reduced LVEF, preexisting Atrial fibrillation and presence of higher degree residual MR following TEER might be high risk of iASD persistence.

All in all, trans-septal pressure gradients and pulmonary vascular resistance are the driving forces for the development of an interatrial shunt, especially elevated left heart pressure or volume. The correction of functional MR by means of TEER can improve patients’ hemodynamic profile. However, the hemodynamic relevance of the residual iASD is still debated. Mandurino-Mirizzi et al^[17]^ performed the right heart catheterization to monitor the hemodynamic changes of the patients at baseline after general anesthesia induction and at the end of TEER. The data suggested that the acute hemodynamic changes after TEER were not influenced by the induction of iASD in patients. Meanwhile, it revealed that the Mitraclip positioning was followed by a significant immediate improvement in cardiac output, mean pulmonary artery pressure, pulmonary artery wedge pressure and pulmonary vascular resistance compared with baseline. The study demonstrated that elevated left atrium pressure after TEER was associated with persistence of iASD at 1 month and 12 months after the procedure.^[10]^ These effects are likely to contribute to a significantly reduced LVEF in iASD patients compared to non-iASD patients during follow-up monitoring.

### 4.2. Hemodynamic consequences of IASD and clinical outcome

During the TEER procedure, the surgeon introduces the device into the femoral vein and then delivers the device to the right atrium, next requires a puncture of the atrial septum from the RA to LA, resulting in an iASD with a concomitant reduction of flow volume across the mitral valve, which usually closes spontaneously in the later stage and only causes a transient and generally negligible shunt. Although significant shunt is a rare complication, but accurate risk stratification of iASD is necessary to screen for significant left-to-right shunt and avoid patients developing progressive heart failure. These patients require close follow-up and periodic monitoring by echocardiography to keep up with the impacts on hemodynamics of persistent iASD after TEER. There is a lack of relevant research about acute hemodynamic and clinical instability due to significant shunt. Until now, only a few data are available about the hemodynamic consequences and clinical effects of persistent iASD. Echocardiography such as transesophageal echocardiography (TEE) or transthoracic echocardiography may be routinely used to assess the defect size of persistent iASD in specific patients with symptoms of RV dysfunction after TEER.^[18]^ We have limited information from TEE or transthoracic echocardiography alone and need more means to monitor hemodynamic changes during follow-up, the problem of hemodynamic relevance is difficult to figure out without proper invasive or noninvasive quantitative hemodynamic monitoring.^[18]^ Besides hemodynamic instability as a result of acute RV dysfunction, the procedure of TEER mediates a significant decompression of the left atrium through the IASD and may be beneficial for selected patient populations. Some studies described acute hemodynamic changes after a TEER procedure and found an increase in cardiac output and a reduction in systemic as well as pulmonary vascular resistance.^[18,19]^ There are hints that suggest that iASD persistence may be beneficial for heart failure patients by relief of left atrial pressure and volume overload. Indeed, clinical studies that analyzed the positive effects of deliberately created interatrial left-to-right shunts in patients with HF, irrespective of the preserved^[20]^ or reduced^[21]^ left ventricular ejection fraction thereby resulted in the new therapeutic technique of interatrial shunt device.^[22,23]^ Nevertheless, Patients with symptoms of reduced LVEF, Atrial fibrillation, residual higher degree MR and large shunt of iASD after TEER might more possible to suffer from the right heart dysfunction and hemodynamic instability.^[24,25]^ It showed that right heart diameter increased in patients with persistent iASD in our meta-analysis. Simultaneously, there was no significant difference in right ventricular systolic pressure between + IASD patients group and non-iASD patients group, as described in previous studies.^[24]^

Rehospitalization rates for heart failure were described in 5 studies, Patients with iASD persistence after TEER had a great risk of HF rehospitalization compared to non-iASD patients, indicating worse clinical outcomes. There was no significant difference in overall mortality at follow-up period between the iASD patients and non-iASD patients, as well as stroke/TIA rates, the outcome data about stroke/TIA were rare and only described in two studies. Due to these contradictory considerations and lack of the hemodynamic consequences of an iASD with clinical outcome data, it’s necessary to conduct more and larger clinical studies in independent patient cohorts to address the remaining questions.

### 4.3. Whether and when to close the iASD

To the best of our knowledge, the procedure of TEER usually result in a negligible shunt. A successful procedure does not generally induce right ventricular overload and complications of hemodynamic instability are scarce. In theory, an iASD could provoke paradoxically negative effects and neutralize some of the positive effects of the TEER procedure by RV overload. Hoffmann et al^[26]^ suggested that the beneficial aspect of TEER is at least partly attributable to the creation of an iASD. This might be secondary to lower left atrial pressure and reduced LV filling, which in turn could reduce MR. In a single-center study, patients with an iASD presented with signs and symptoms of heart failure that were attenuated at 1-month follow-up after closure. This could be a consequence of the volume shift from the RV to the LV with reduced pulmonary but increased systemic cardiac index and favorable biventricular interaction at maintained LV filling pressure after iASD closure.^[27]^ Javed et al^[28]^ described sudden increase in left atrial pressures with the risk of acute pulmonary edema following closure of congenital ASDs. It underlined the importance of thoughtful hemodynamic evaluation of patients scheduled for iASD closure, and the implications of iASD closure on the consequence of TEER deserved some considerations. Therefore, there are also reports that advocate for a cautious explanation regarding the hemodynamic consequences of an iASD after TEER in specific patient populations.^[29]^ In the interim, closure of iASD after TEER is not routinely performed, it revealed that iASD closure was not superior to conservative therapy after TEER in the MITHRAS trial.^[30]^ The finding of Lurz et al^[31]^ given rise to the question whether iASD closure at 1-month after TEER might have been too early to differentiate patients who might benefit from closure as opposed to those patients with a certain likelihood of shunt reduction over time. In this randomized controlled trial involved patients with persistent iASD one month after TEER, iASD closure did not improve functional or clinical midterm outcomes, such as the 6-minute walk test distance, NT-proBNP, death or hospitalization for HF. To date, data on iASD management are sparse, and there is currently no guideline recommendation established. The decision of whether and when to conduct the iASD-closure is a challenging one.^[32]^ There are some evidences that generally advocates closure of an IASD after a TEER procedure, the consequences of iASD may become more manifest in clinical practice. Immediate closure of iASD should be considered only when the hemodynamic inability because of shunting at the atrial level and the patient’s hypoxia or multiple comorbidities, including poor pulmonary function, right-to-left shunting, significant tricuspid regurgitation and preexisting right heart dysfunction. These patients should be identified before the procedure to portend a poor clinical consequence from the iASD.^[32]^ In the era of vigorous development for treatment with TEER, although significant shunt is a rare complication, but accurate risk stratification of iASD is necessary to screen for significant left-to-right shunt and avoid patients developing progressive heart failure. These patients require close follow-up and periodic monitoring to keep up with the impacts on hemodynamics of persistent iASD and more clinical studies are necessary to suggest guidelines for the treatment of iASDs.

## 5. Limitations

In this meta-analyze, there are several limitations. It should not be neglected that the majority of studies included were retrospective studies accompanied by their intrinsic limitations. Therefore, we can’t exclude a relevant selection bias and that limiting the stringency of our findings. The timing of follow-up examinations varied between different studies and reduced the credibility of the morbidity of iASD in various studies. Furthermore, the data of TEE were not available in all studies, only parts of studies included used TEE to detect iASD during the follow-up examinations. So, the diameter of iASD that might be too small to be overlooked and the morbidity of iASD might be underestimated.^[33]^ Taking into account the limitations of this meta-analyze, it is necessary to carry out more prospective multicenter trials to understand the impacts on hemodynamics in patients of iASD persistence. However, this meta-analysis made a comprehensive summary and pointed out the existing problems in the current research in the field of TEER. It’s helpful for us to plan meaningful high-quality researches.

## 6. Conclusion

In summary, there is no compelling evidence that generally advocates closure of an IASD after a TEER procedure. Furthermore, successful TEER procedure usually does not induce a hemodynamic relevant interatrial left-to-right shunt. Some case reports described several related factors that may predict iASD persistence following TEER, possibly leading to right heart failure. there is no large clinical study available that clearly identified patients’ outcome or risk factor prediction model of iASD until now. Our study has some limitations that require cautious interpretation of the results. In order to generalize our findings, it is necessary to carry out more high-quality trials and multicenter larger studies to make in-depth exploration of the impacts on hemodynamics in patients of iASD persistence. What’s more, it’s meaningful to perform a close follow-up and periodic monitoring through a noninvasive and convenient method. Furthermore, we look forward to a guideline recommendation established for the treatment of iASDs so as to make a decision of whether and when to conduct the iASD-closure at the appropriate time.

## Author contributions

**Data curation:** Deng Lei.

**Formal analysis:** Deng Lei.

**Funding acquisition:** Baoshi Zheng.

**Methodology:** Deng Lei, Chunlan Zhou, Chen Luo.

**Project administration:** Chunlan Zhou, Chen Luo.

**Supervision:** Junqing Zhou, Baoshi Zheng.

**Validation:** Junqing Zhou, Chen Luo, Baoshi Zheng.

**Writing – original draft:** Deng Lei.

**Writing – review & editing:** Junqing Zhou, Baoshi Zheng.
